# Dynamics of evolutionary succession and coordination between opposite adaptations in cuckoo hosts under antagonistic coevolution

**DOI:** 10.1038/s42003-024-06105-9

**Published:** 2024-04-03

**Authors:** Canchao Yang, Ziqi Zhang

**Affiliations:** Ministry of Education Key Laboratory for Ecology of Tropical Islands, College of Life Sciences, Hainan Normal University, Haikou, 571158 China

**Keywords:** Animal behaviour, Coevolution

## Abstract

Adaptations are driven by specific natural selection pressures throughout biological evolution. However, these cannot inherently align with future shifts in selection dynamics, thus manifesting in opposing directions. We performed field experiments on cuckoo hosts to investigate the coexistence and conflict between two evolutionarily successive but opposing behavioral adaptations—egg retrieval and rejection. Our findings provide key insights. (1) Egg rejection against brood parasites in hosts reshapes egg retrieval to flexible reactions—retrieval, ignoring, or outright rejection of foreign eggs outside the nest cup, departing from instinctual retrieval. (2) Parasitism pressure and egg mimicry by parasites remarkably alter the proportions of the three host reactions. Host species with higher parasitism pressure exhibit frequent and rapid rejection of non-mimetic foreign eggs and reduced ignoring or retrieval responses. Conversely, heightened egg mimicry enhances retrieval behaviors while diminishing ignoring responses. (3) Cuckoos employ consistent mechanisms for rejecting foreign eggs inside or outside the nest cup. Direct rejection of eggs outside the nest cup shows that rejection precedes retrieval, indicating prioritization of specific adaptation over instinct. (4) Cuckoo hosts navigate the conflict between the intentions and motivations associated with egg rejection and retrieval by ignoring foreign eggs, a specific outcome of the rejection–retrieval tradeoff.

## Abstract (web summary)

This study revealed that cuckoo hosts coordinated two evolutionarily successive but opposing behaviours by reshaping the instinctive egg retrieval reaction to different flexible reactions during their coevolution with parasitic cuckoos.

## Introduction

Biological evolution involves changes in organism population properties that transcend the lifetime of a single individual^1^. Such changes are adaptations wherein organisms interact with various factors, including conspecifics, other species, and the environment^2,3^. Natural selection changes in direction and intensity over time, and therefore, does not always remain constant^4^. Consequently, a current adaptation cannot predict or guarantee its adaptability in the future during evolution. For example, the well-known blind spot in the human eye results from a conflict between optic nerve conduction and the inner lining of the retina^5^. The blind spot provides evidence that an adaptation cannot predict consequences but evolves based on previous events, even if they are contradictory. Behavior is the forerunner of evolution, but studying different behavioral adaptations along an evolutionary trajectory is difficult due to the lack of fossil or anatomical evidence^6,7^. However, egg retrieval and rejection behaviors in birds may provide a testable case for such studies.

Egg retrieval behavior refers to the act of an incubating bird retrieving eggs that have accidentally rolled out of the nest. This behavior is believed to be a common ancestral behavior among ground-nesting birds that have not evolved elegant nest knitting^8^. As egg Retrieval cannot be suspended after stimuli until the bird completes the entire process, the behavior is regarded as a fixed pattern of instinctive behavior^8,9^. In contrast, egg rejection is a more recent and specific adaptation that has evolved as a defense against brood parasitism^10,11^. Egg rejection is flexible and can be enhanced via learning and social transmission^12–14^. Notably, the motivation and intention for egg rejection oppose those of egg retrieval; the former refers to recognizing and rejecting eggs that are inside nests, whereas the latter refers to distinguishing and retrieving eggs that are outside nests. Therefore, these two behaviors are successive adaptations on an evolutionary axis related to different levels of egg discrimination, but the natural selection direction for behavioral intention is the opposite. For egg retrieval, the birds need to distinguish eggs from non-egg-shaped objects, while for egg rejection, they need to identify parasite eggs from their own eggs. Consequently, common hosts of brood parasites face and cope with the conflict between egg retrieval and rejection when they possess both behavioral adaptations because when confronted with an egg outside the nest cup, the former will stimulate the birds to retrieve it whereas the latter will trigger them to reject it. Studies on the relationship between these two behaviors will contribute to revealing the evolutionary and maintained mechanisms between two successive but opposing adaptations.

**Fig. 1:**
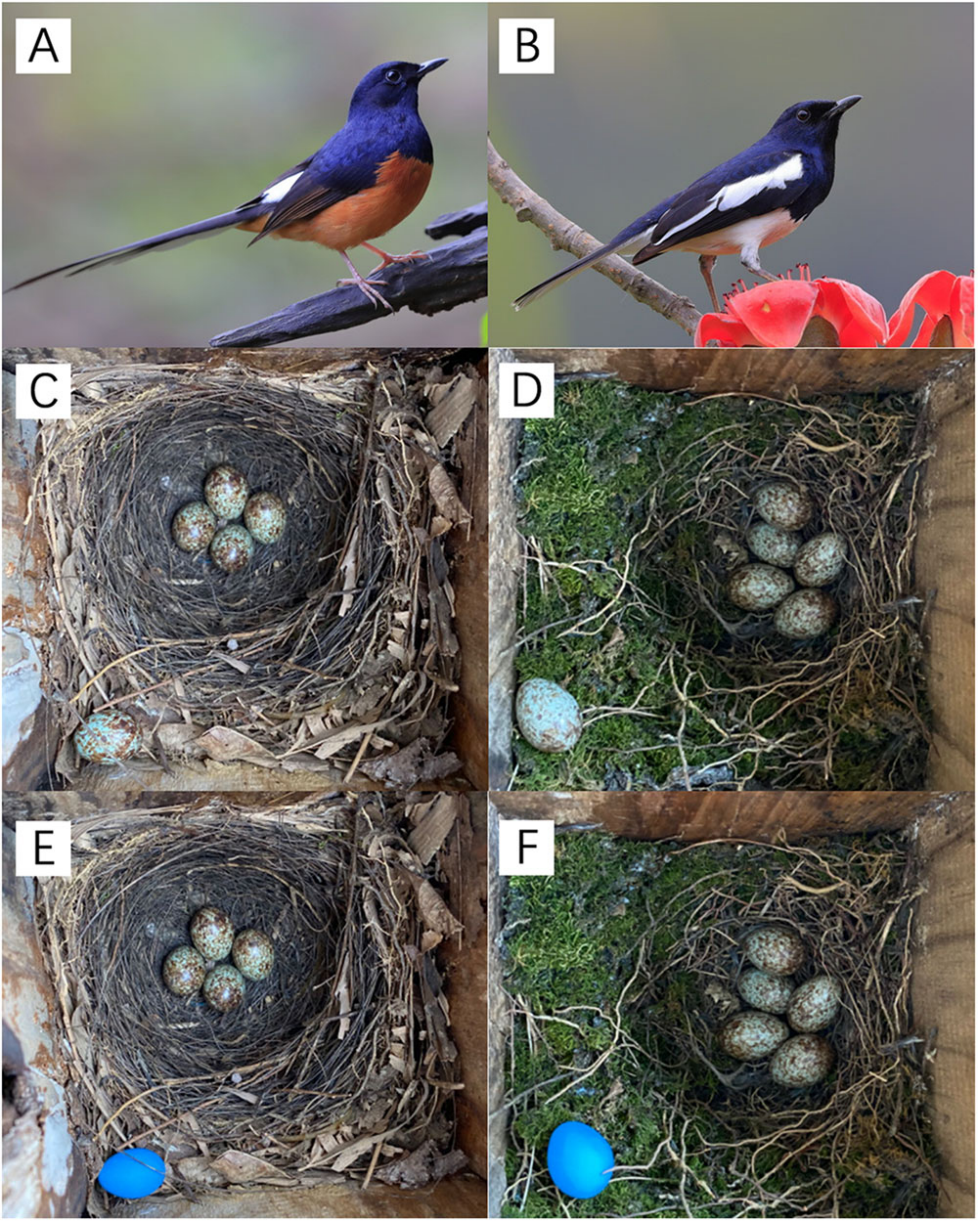
Images of the studied species and examples of experimental treatment with a manipulated egg in the ONC (outside the nest cup) treatment. **(A)** Male white-rumped shama; (**B**) male Oriental magpie-robin; (**C**) shama nest with a manipulated conspecific egg in ONC treatment; (**D**) magpie-robin nest with a manipulated conspecific egg in ONC treatment; (**E**) shama nest with a manipulated model egg in ONC treatment; (**F**) magpie-robin nest with a manipulated model egg in ONC treatment. **A**, **B** were photographed by Li Cheng with permission.

**Fig. 2:**
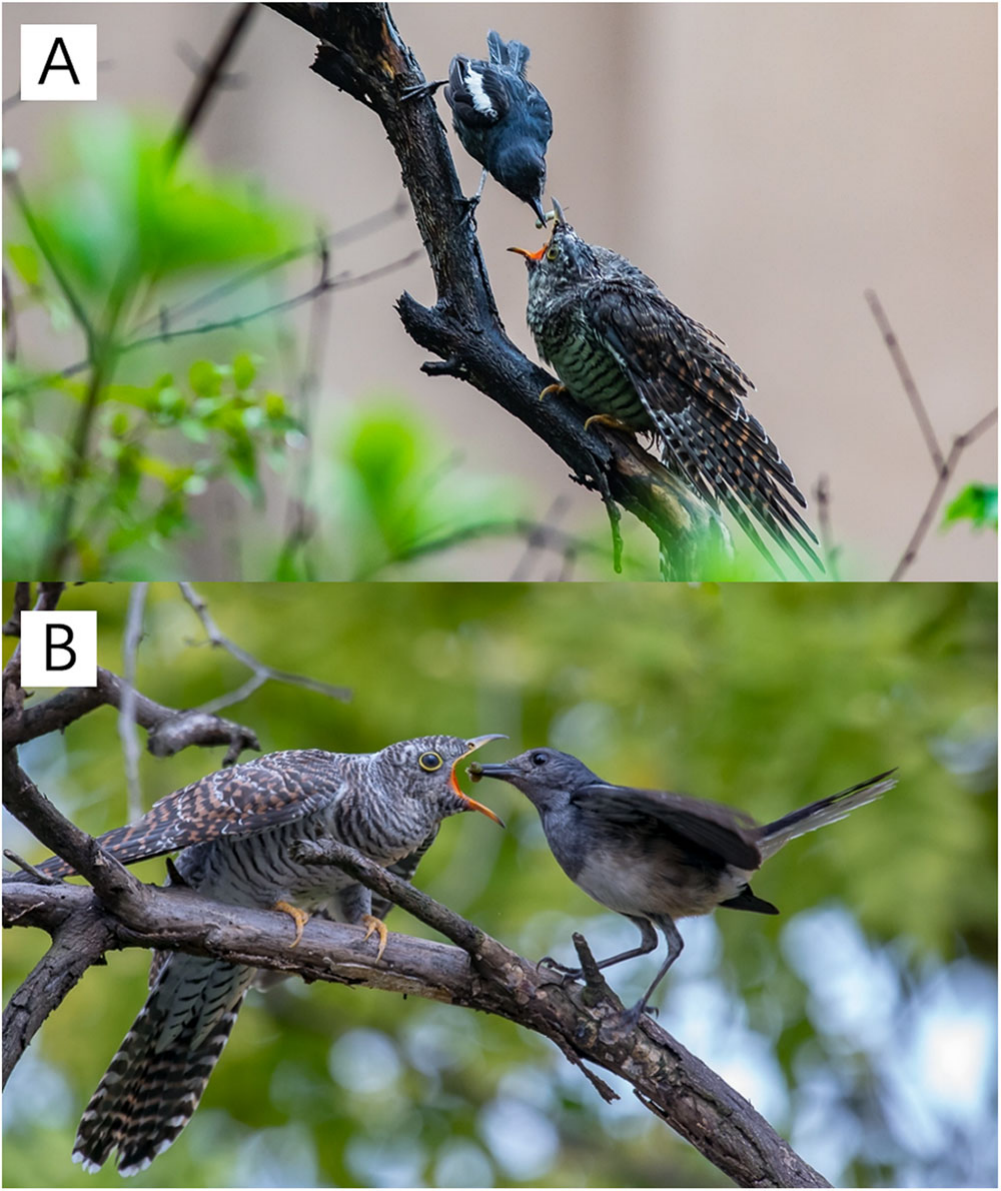
Images of Oriental magpie-robins feeding common cuckoo chick. **A** Male magpie-robin feeding cuckoo chick; **B** female magpie-robin feeding cuckoo chick. The images were photographed by Guoqiang Yu with permission.

Despite evidence of the conflict between egg retrieval and rejection, studies on the topic are limited^15^. Approximately 100 species of obligate brood parasites exist globally^16^, including the famous parasitic cuckoos (*Cuculidae spp*.) and cowbirds (Icteridae spp.), all of which are altricial birds (except for *Heteronetta atricapilla*, an obligate parasitic duck). However, only two studies on egg retrieval behavior have been conducted in altricial birds^15,17^, of which only one focused on the relationship between egg retrieval and rejection^15^. As the studied species have not been exploited by any parasitic species, their relationship with possible brood parasites is unclear^15^. In this study, we conducted a field experiment^18^ to study and compare the behavioral relationship between egg retrieval and rejection in two closely related sympatric breeding species: the Oriental magpie-robin (*Copsychus saularis*) and white-rumped shama (*C. malabaricus*) (Fig. 1A, B). The studied population of magpie-robins was exploited by the common cuckoo (Fig. 2), whereas the shamas were not parasitized by any of the cuckoo species.

This study aimed to determine the effect of two natural selection aspects (cuckoo parasitism and host defense) on two opposite behavioral adaptations (egg retrieval and rejection) and how hosts cope with the conflict between these adaptations. Furthermore, the pattern of egg rejection and retrieval reactions is expected to change according to the selection intensity of cuckoo parasitism and host defense. Specifically, magpie-robins are expected to reject more foreign eggs than shamas, whereas both species should reject more non-mimetic eggs than highly mimetic foreign eggs.

## Methods

### Study sites and species

This study was performed in the Nonggang National Nature Reserve, Guangxi Zhuang Autonomous Region, Southwestern China, during the breeding seasons (i.e., April–July of the two study years, 2021–2022). The study area is located in the Sino-Vietnamese border region (22°13′N, 106°42′E), a typical limestone area located in the northern margin of the tropics at an altitude between 150 and 650 m. Mean annual rainfall and temperature in this region are 1150–1550 mm and 20.8–22.4 °C, respectively^19^. Nest boxes were used to provide nest sites for the two studied species, the Oriental magpie-robins and white-rumped shamas, which breed in sympatry in the study area, with similar nest and egg phenotypes (Fig. 1). The species are closely related within the genus *Copsychus*, according to the taxonomic information presented in “Birds of the World” from the Cornell Lab of Ornithology^20,21^. Magpie-robins are a common host for the common cuckoo^22^, whereas the parasitic host status of shamas is unclear. However, it is assumed that shamas are a potential host for cuckoos^21^. The studied magpie-robin population was confirmed to be exploited by the common cuckoo (Fig. 2), unlike the shama population. However, the magpie-robin parasitism rate was unknown, as the hole entrances in the nest boxes used to perform an experiment may have been too small (6 cm in diameter) for a cuckoo entrance. Although no parasitism was found in the nest boxes, cuckoo fledglings fed by magpie-robins from natural nests were frequently observed in the images (Fig. 2). Thus, we predicted that magpie-robins experienced a higher degree of selection intensity from cuckoo parasitism than that experienced by shamas. We conducted artificial parasitism to confirm this prediction by testing the egg recognition capacity of these two species (see the field procedure for the first treatment below for details). Additionally, the findings of our daily investigation during the egg-laying stage showed no signs of intraspecific parasitism in the magpie-robin or shama nests. Notably, using egg recognition capacity rather than parasitism rates to represent parasitism pressure was more feasible in this study for several reasons: (1) the egg recognition capacity would reflect the intensity of interaction between the hosts and parasites^23,24^; (2) parasitism rates may be underestimated or overestimated owing to different levels of egg rejection in hosts^25,26^; and (3) egg rejection rates were expected to differ between the two studied species and, thus, using egg recognition capacity as a representation of parasitism pressure ensured greater comparability among species.

### Experiment on egg recognition capacity

To investigate the egg recognition capacity of the studied species, we conducted an experiment simulating artificial parasitism during the first study year. The experimental protocol termed the “inside nest cup (INC)” treatment involved the introduction of a model egg (identical in size to that of the host egg) into the nest cup to replace one of the authentic host eggs. The model egg was crafted from blue polymer clay to mirror the prevailing phenotype of common cuckoo eggs in China, given that magpie-robins are documented to be parasitized by common cuckoos using blue eggs^22^. The treatment was performed the day after the hosts completed their clutches (magpie-robins: *n* = 39, excluding one predation case; shama: *n* = 35). Subsequently, the manipulated clutches were monitored daily for 6 days to confirm the hosts’ responses, which were classified into acceptance (model eggs incubated by the hosts), ejection (model eggs ejected by the hosts), or desertion (clutches deserted by the hosts). A control trial was used to control for manipulation disturbances (*n* = 30 for magpie-robins and shamas), in which host clutches followed the same investigation procedure as the INC treatment without model egg replacement. No desertion or other abnormal phenomena were observed in the control trials; therefore, both ejection and desertion were regarded as rejections by the hosts. The model egg in this treatment was non-mimetic to the host egg. Thus, the recognition capacity in this study referred to host recognition based on non-mimetic eggs. Here, we used the egg rejection rate to represent egg recognition capacity. To avoid pseudo-replication, the time between the first and last experimental nests was limited to approximately 37 days according to the reproductive cycle (i.e., from nest building to fledging) of the hosts^20,27^.

### Experiment on egg rejection and retrieval reaction patterns

To investigate the relationship between the reaction patterns associated with egg rejection and retrieval, we carried out the “outside nest cup (ONC)” treatment. This treatment encompassed two groups of trials, each involving the replacement of either one model or one conspecific egg instead of one of the clutch eggs. At the same time, the substituted egg was positioned on the nest platform outside the nest cup, at a distance of 2 cm from the rim of the nest cup (Fig. 1C–F). The timing of the manipulation and investigation procedures were the same as those in the INC treatment. The host responses in ONC treatment were classified into retrieval (the eggs outside the nest cups were retrieved and brought back into the nest cups by the hosts), ejection (the eggs outside the nest cups were ejected by the hosts), ignoring (the eggs outside the nest cups were neither retrieved nor ejected by the hosts), or desertion (the hosts deserted the clutches). Some observed nests were randomly selected (*n* = 27) to monitor the behavioral reaction using a mini camera (WJO3, Hisilicon, Shenzhen, Guangdong, China). Additionally, control trials (*n* = 25 for both studied species) were conducted to control for manipulation disturbances, and no desertions or other abnormal phenomena were observed.

### Classification of reactions and mimetic levels

**Fig. 3:**
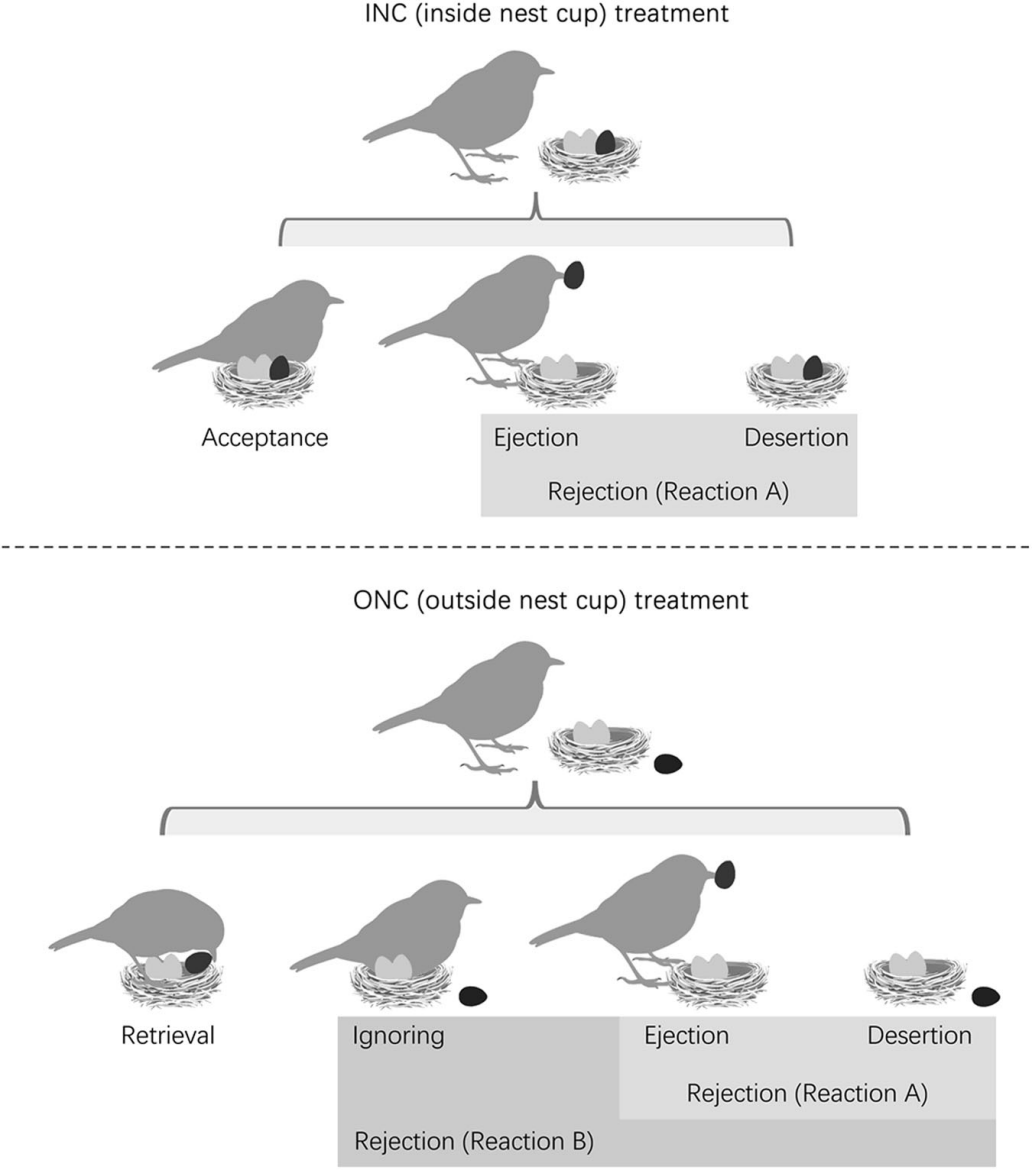
A schematic diagram illustrating the classification of reactions in this study. The black egg refers to the experimental egg with manipulation. In the INC treatment, the black egg refers to a model egg, while in the ONC treatment, the black egg refers to a model or conspecific egg. Figure created in PowerPoint.

Two rejection reactions were identified during the treatment: Reaction A, in which ejection and desertion were combined as rejections (applicable to INC and ONC treatments), and reaction, B which included the ignoring response as rejection, as this behavior could be construed as a mode of rejection wherein hosts abstain from retrieving the eggs (only applicable for ONC treatment). We used reactions A or B to analyze the results because ignoring cannot be easily categorized. For the accepting reaction of INC, the hosts would incubate the eggs without recognition. For the rejecting reaction, the hosts would directly reject the eggs after recognition. The ignoring reaction, however, resembled an undecided process. Using the classification of reaction A or B to analyze the hosts’ responses to different mimetic levels of parasite eggs would help us reveal the role of the ignoring reaction. Therefore, reactions A and B did not differ for the INC treatment yet diverged within the context of the ONC treatment (Fig. 3). Notably, conspecific eggs within the conspecific group of the ONC treatment were randomly selected from the same clutches to mitigate the potential influence of inter-female variation in egg phenotypes. Therefore, the model and conspecific egg groups in ONC treatment represent non-mimetic and highly mimetic eggs, respectively. Additionally, each experimental nest of ONC treatment received only one egg type (either a model or conspecific egg), while both the magpie-robin and shama groups received two egg types (magpie-robin: *n* = 24 for the model group excluding one predation case, *n* = 24 for the conspecific group; shamas: *n* = 23 for the model group, *n* = 25 for the conspecific group).

### Two aspects of natural selection

Using the aforementioned experimental design, we simulated a situation based on two aspects of natural selection: Egg recognition capacity and foreign egg mimicry. Egg recognition capacity is selected by, and thus reflects the intensity of, cuckoo parasitism (one aspect of natural selection) and was divided into two levels according to the two studied species (magpie-robin and shama). Although egg recognition capacity may persist owing to past selection, it reflects the intensity of interaction with parasites^28,29^. However, this will not influence our study because we did not focus on past or present selection but rather aimed to determine the effect of such intensity changes on host behavior. Foreign egg mimicry reflects the intensity of host defense (another aspect of natural selection) because stronger host defense will promote the parasites to evolve more optimized egg mimicry. Foreign egg mimicry was divided into two levels: non-mimetic and highly mimetic. This allowed us to study the relationship between egg retrieval and rejection under two aspects of natural selection that were related yet different. Egg recognition capacity represents the intensity of encountering parasites whereas egg mimicry reflects the levels of cognition. The egg recognition capacity can be studied under different levels of egg mimicry whereas the same level of egg mimicry can lead to different capacities of egg recognition between hosts.

### Statistics and reproducibility

Four Markov chain Monte Carlo technique–generalized linear mix models (MCMC-GLMM) were built and used to investigate the effects of experimental manipulation on host reactions. In the first two models, the response variable was either reaction A or B to the model egg, while the fixed effects were species (magpie-robin or shama), treatment (INC or ONC treatment), clutch size, and laying date (of the first egg). In the second two models, the response variable was either reaction A or B of ONC treatment, and the fixed effects were group (model or conspecific egg), species, clutch size, and laying date. Nest identity was a random effect in all models. The MCMC-GLMM calculates the posterior estimate using Bayesian analyses, which provide a posterior mean and 95% credible interval^30^. Cox regression models were used to analyze the reaction time associated with egg rejection or retrieval. This analysis was performed by incorporating both the incidence of a rejection or retrieval event and its latency (i.e., the timing of the daily investigation) into the survival function. As the Cox model assumes a consistent shape for the survival function, we imposed constrained time intervals of investigation in this study that spanned six days with daily frequency^31^. In instances where no occurrence transpired during the six-day window, a latency period of six days without an event was encompassed in the Cox models as a censored value. Furthermore, Kaplan–Meier curves of survival probability were established to illustrate the significant results derived from the Cox models. MCMC-GLMM, Cox regression, and Kaplan–Meier curves were generated using the packages *MCMCglmm*, *survminer*, and *survival*, respectively, in R (v. 4.2.2) for Windows (R Foundation for Statistical Computing, Vienna, Austria).

### Reporting summary

Further information on research design is available in the Nature Portfolio Reporting Summary linked to this article.

## Results

### Reaction to model egg in INC/ONC treatment

**Fig. 4:**
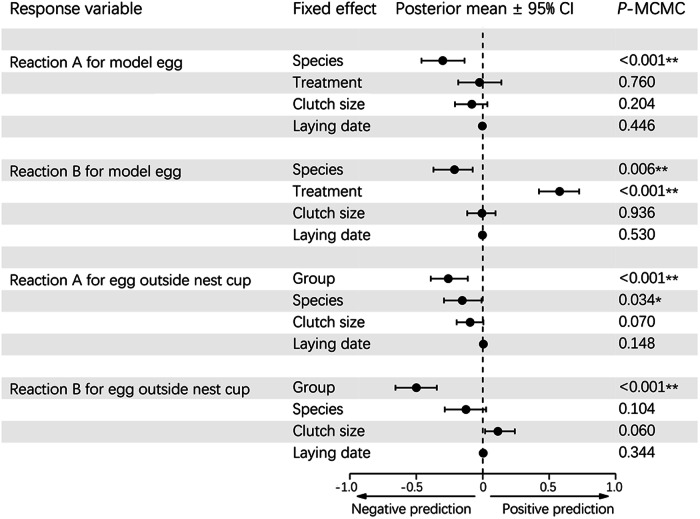
Results of generalized linear mixed models using Markov chain Monte Carlo techniques. Ignoring behavior was excluded from reaction A but included in reaction B as part of rejection. Species: magpie-robin or shama; treatment: INC (inside nest cup) or ONC (outside nest cup); group: conspecific or model egg. The nest identity was a random effect in this model. Here, 95% CI refers to the 95% credible interval in which the prediction significance was higher when the values of the 95% CI were further away from zero.

**Fig. 5:**
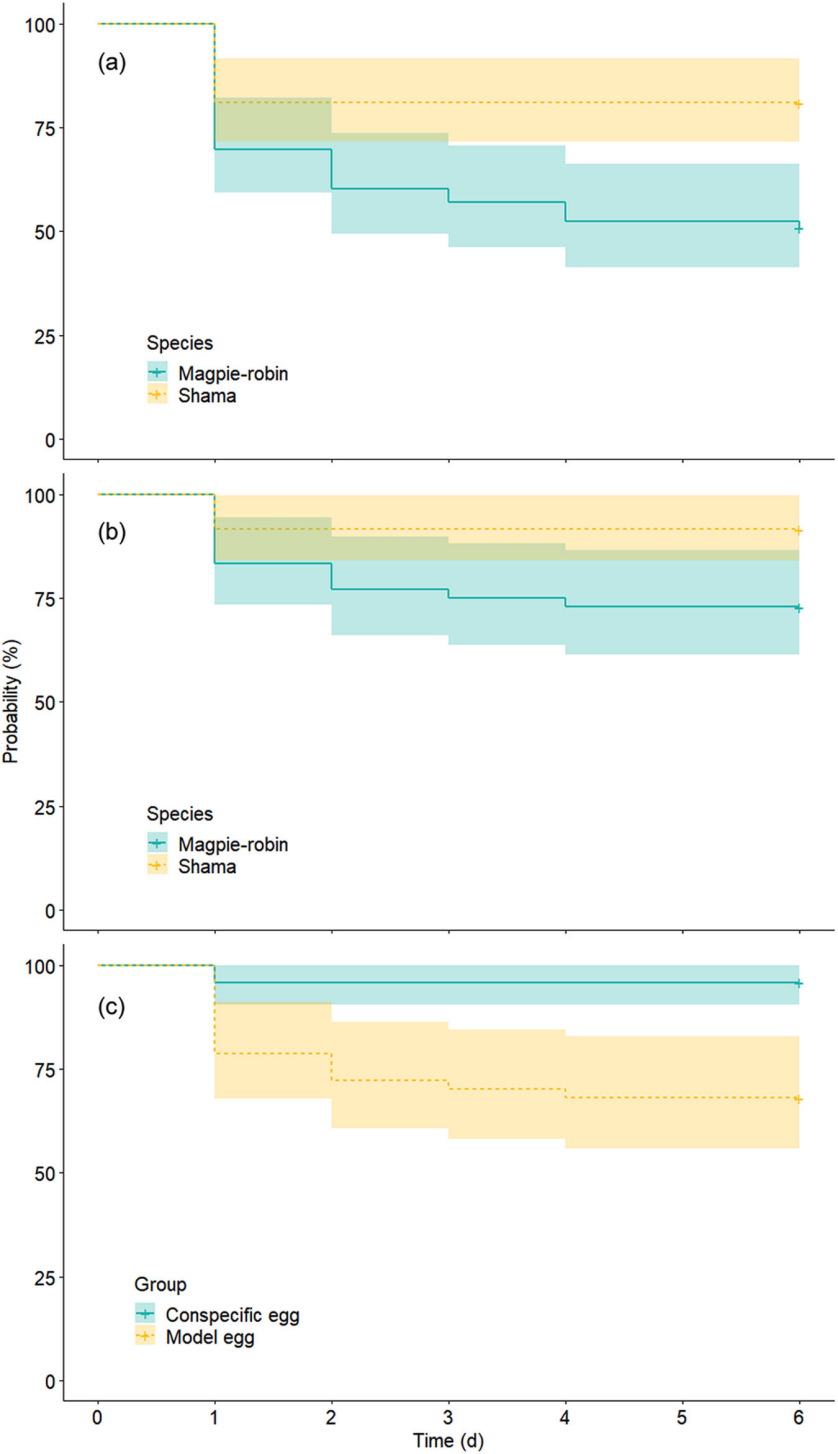
Kaplan–Meier survival curves illustrating the significant results of the Cox regression model in Table 2. **a** rejection reaction to model egg between species; (**b**) rejection reaction in ONC (outside nest cup) treatment between species; (**c**) retrieval reaction in ONC treatment between conspecific and model eggs. The shaded region indicates a 95% confidence interval, and the crossover symbols on the 6th day refer to the censored values.

The videos revealed that both the magpie-robins and shamas used their bills to eject or retrieve eggs, and all ejections were performed via grasping. Magpie-robins exhibited a rejection rate of 48.72% in response to the blue model eggs in the INC treatment, comprising 84.21% ejections and 15.79% desertions (Table 1). In comparison, shamas rejected 22.86% of the blue model eggs, which was less than half the rejection rate of magpie-robins. Ejection and desertion accounted for 75% and 25% of the rejection cases, respectively. The MCMC-GLMM indicated that the model egg rejection rate of magpie-robins was significantly higher than that of shamas (Species: posterior mean = −0.3, 95% CI = −0.46 to −0.14, *P* < 0.001, MCMC-GLMM; Fig. 4). Notably, the treatment did not predict model egg rejection (Fig. 4); both magpie-robins and shamas displayed a similar proportion of model egg rejection in the INC treatment relative to that in the ONC treatment (magpie-robin: 48.72% vs. 50%; shama: 22.86% vs. 13.04%; Table 1). Furthermore, neither clutch size nor egg-laying date predicted the egg rejection rate (Fig. 4). However, when the ignoring reaction was considered as a form of rejection, both species and treatment significantly predicted the model egg rejection rate (Species: posterior mean = −0.21, 95% CI = −0.37, −0.08, *P* = 0.006; Treatment: posterior mean = 0.58, 95% CI = 0.42, 0.73, *P* < 0.001; MCMC-GLMM). In these cases, model egg rejection rates in the ONC treatment reached 100% and 86.96% for magpie-robins and shamas, respectively (surpassing the 48.72% and 22.86% rejection rates, respectively), observed in the INC treatment (Table 1). When considering the latency to rejection, the reaction time to model eggs of magpie-robins was significantly shorter than that of shamas (Z = −3.072, *P* = 0.002, Cox regression; Table 2; Fig. 5).

**Table 1:** A summary of the experimental data in this study (numbers in brackets refer to the percentage)

Treatment	Group	Species	Acceptance	Ejection	Desertion	Ignoring	Retrieval	*N*	Rejection in Reaction A	Rejection in Reaction B
INC	Model egg	Magpie-robin	20 (51.28)	16 (41.03)	3 (7.69)	N/A	N/A	39	19 (48.72)	19 (48.72)
		Shama	27 (77.14)	6 (17.14)	2 (5.71)	N/A	N/A	35	8 (22.86)	8 (22.86)
ONC	Model egg	Magpie-robin	N/A	9 (37.5)	3 (12.5)	12 (50)	0 (0)	24	12 (50)	24 (100)
		Shama	N/A	3 (13.04)	0 (0)	17 (73.91)	3 (13.04)	23	3 (13.04)	20 (86.96)
	Conspecific egg	Magpie-robin	N/A	0 (0)	1 (4.17)	10 (41.67)	13 (54.17)	24	1 (4.17)	11 (45.83)
		Shama	N/A	0 (0)	1 (4)	9 (36)	15 (60)	25	1 (4)	10 (40)

*INC* inside nest cup, *ONC* outside nest cup, *N/A* Not applicable.

**Table 2:** Results of Cox regression models investigating the reaction time of egg rejection or retrieval by incorporating its occurrence and latency

Model	Response variable	Fixed effect	Coefficient	S.E.	*Z*	*P*
1	Rejection	Treatment	−0.137	0.322	−0.426	0.670
		Species	−1.080	0.352	−3.072	0.002**
2	Rejection	Group	2.221	0.754	2.947	0.003**
		Species	−1.289	0.573	−2.251	0.024*
3	Retrieval	Group	0.172	0.267	0.644	0.520
		Species	0.102	0.250	0.407	0.684

Mode 1: rejection of model egg, treatment: INC (inside nest cup) or ONC (outside nest cup), species: magpie-robin or shama; Model 2: egg rejection of ONC treatment, group: conspecific or model egg; Model 3: egg retrieval of ONC treatment.

*P < 0.05; **P < 0.01.

### Reaction to conspecific or model egg in ONC treatment

For conspecific eggs in ONC treatment, only 4.17% and 4% of the magpie-robin and shama nests, respectively, exhibited rejection reactions, and all rejection cases were executed by desertion (Table 1). These rejection rates were significantly lower than those for model eggs in ONC treatment (Group: posterior mean = −0.26, 95% CI = −0.39, −0.11, *P* < 0.001; MCMC-GLMM) and differed between species (Species: posterior mean = −0.15, 95% CI = −0.29, −0.01, *P* = 0.034; MCMC-GLMM; Fig. 4). In contrast, if the ignoring reaction was considered rejection, the group but not the species reached significance in terms of predictions (Group: posterior mean = −0.5, 95% CI = −0.66, −0.35, *P* < 0.001; Species: posterior mean = −0.13, 95% CI = −0.29, 0.03, *P* = 0.104; MCMC-GLMM; Fig. 4). In this instance, the rejection rates of conspecific eggs in ONC treatment were similar between magpie-robins (45.83%) and shamas (40%), in which the ignoring reactions accounted for 90.91% and 90% of the rejection cases, respectively. Correspondingly, the retrieval rates of conspecific eggs in ONC treatment were similar at 54.17% and 60% in magpie-robins and shamas, respectively (Table 1). Nevertheless, for the model eggs in ONC treatment, none of the magpie-robins exhibited a retrieval reaction (0%), whereas the shamas retrieved 13.04% of the model eggs (Table 1). Finally, when accounting for latency to rejection, the reaction time to the model egg proved notably shorter than that observed for the conspecific egg (*Z* = 2.947, *P* = 0.003, Cox regression; Table 2; Fig. 5). Moreover, the shamas exhibited a slower reaction time than that exhibited by the magpie-robins (*Z* = −2.251, *P* = 0.024, Cox regression; Table 2; Fig. 5). Notably, the reaction time for egg retrieval did not differ between groups or species (Table 2).

## Discussion

In both treatments, the number of non-mimetic model eggs rejected by magpie-robins was higher than that rejected by shamas. Magpie-robins rejected half of the non-mimetic model eggs in the INC and ONC treatments (48.72% and 50%, respectively) and did not retrieve them in the ONC treatment. In comparison, shamas only rejected 22.86% and 13.04% of the model eggs in INC and ONC treatments, respectively, and retrieved 13.04%. Furthermore, the reaction time required to reject model eggs was significantly shorter in magpie-robins than in shamas. These results are consistent with our predictions, confirming that (1) the magpie-robins were under a higher intensity of cuckoo parasitism, (2) the magpie-robins were more aggressive than shamas in excluding foreign eggs but more unwilling to retrieve them because more intensive cuckoo parasitism led to selection for stronger egg rejection as a defensive adaptation, and (3) the shamas evolved a similar defense; however, it was weaker than that of the magpie-robins. In summary, the pattern of egg rejection in these two host species was predicted based on the selection pressures of cuckoo parasitism.

Prior research has posited that birds devoid of evolved egg rejection behavior, such as the graylag goose (*Anser anser*), would typically respond to all egg-shaped objects outside nest cups by retrieving them into their nests^8^. However, our findings serve as empirical validation that birds directly reject foreign eggs rather than retrieving them outside nest cups. This underscores the pivotal role of specific evolutionary adaptations in egg rejection as a countermeasure against brood parasitism, subsequently reconfiguring the ancestral behavior associated with egg retrieval. For both species, the model egg rejection rates remained consistent whether the eggs were placed inside or outside the nest cups, indicating that the mechanism by which the hosts handled foreign eggs exhibits uniformity, irrespective of the eggs’ positioning relative to the nest cup. For host species that have developed egg rejection as an anti-parasite defense, the egg rejection reaction has a prioritized expression compared to that of the egg retrieval reaction. Second, such behavioral patterns were stronger in magpie-robins than those in shamas, implying a quantitative egg rejection effect on egg retrieval based on the different intensities of parasitism pressure.

When the ignoring reaction was included as part of rejection, 100% and 86.96% of the model eggs in ONC treatment were regarded as rejected by the magpie-robins and shamas, respectively. These model egg rejection rates were significantly higher than those in INC treatment, implying that the ignoring reaction is a maladaptation or specific adaptation to the conflict between egg retrieval and rejection. The hosts likely ignored the model eggs because they did not know what course to take under such conditions and the confusion of motivational conflicts. Alternatively, the hosts may adopt an eclectic method as a result of a tradeoff to handle the motivational conflicts. Regardless of the explanation, these results show that a motivational conflict exists between egg retrieval and host rejection behaviors. We suggest that the ignoring reaction is a specific adaptation rather than a maladaptation, as this behavior predictably changes with the two aspects of natural selection we proposed. First, the ignoring reaction to non-mimetic eggs occurred less frequently in magpie-robins (50%) that had stronger egg recognition than that of the shamas (73.91%). When the ignoring reaction is considered rejection, it accounts for 50% and 85% of the rejection cases for the magpie-robins and shamas, respectively. This indicates that the Shamas were confronting higher uncertainty when faced with motivational conflicts because of their weaker egg recognition. Second, the egg retrieval rates for both host species increased while the ignoring rates decreased with egg mimicry (i.e., from model eggs to conspecific eggs). This implies that the ignoring reaction was participating in the coordination to balance motivational conflicts. Finally, these results indicate that the classification of reaction A or B in this study was effective in revealing the role of ignoring reaction in hosts.

Model eggs in the ONC treatment were rejected at higher frequencies and with shorter reaction times than conspecific eggs were. Egg recognition capacity is determined by cuckoo parasitism^23^, whereas egg mimicry in parasites is determined by host defense^32^. Using two distinct levels of egg mimicry, we showed that the hosts were more aggressive toward non-mimetic eggs than toward highly mimetic eggs in ONC treatment, supporting our prediction. Notably, magpie-robins and shamas exhibited similar proportions of retrieval (54.17% and 60%), ignoring (41.67% and 36%), and rejection (4.17% and 4% by desertion, respectively) of the conspecific eggs in ONC treatment. This result indicates that both magpie-robins and shamas could not discriminate between these eggs and those inside nest cups, as they were highly mimetic eggs from the same clutches. The proportion of egg retrieval, ignoring, and rejection between species differed for non-mimetic eggs but became similar for highly mimetic eggs, which further confirms that the proportion of these reactions is a specific adaptation to the balance of motivational conflicts.

In summary, this study elucidated several key insights. First, the evolutionary trajectory of egg recognition and rejection mechanisms has substantially altered the innate egg retrieval behavior of cuckoo hosts. These hosts exhibit a range of responses—retrieval, ignoring, or rejection—when confronted with foreign eggs outside the nest cup, which is a deviation from their instinctual retrieval behavior. Second, our findings highlight that the frequency of these responses correlates with varying degrees of parasitism pressure and egg mimicry. Under heightened parasitism pressure, hosts more frequently and swiftly reject foreign eggs, while the propensity to ignore or retrieve them diminishes. Conversely, superior mimicry of the parasitic egg markedly increases host retrieval actions but reduces their likelihood of ignoring these eggs. Third, our findings indicate a consistency in the host’s processing mechanism for foreign eggs, irrespective of their placement inside or outside the nest cup, with rejection rates remaining consistent across different positions. However, a notable prioritization of rejection over retrieval responses was observed. Finally, the ignoring reaction shown by hosts can be interpreted as a strategic compromise, navigating the conflicting internal drives between egg rejection and retrieval.

Therefore, we provide vital evidence that the egg retrieval and rejection behaviors that are successive but opposing adaptations on the same evolutionary trajectory can create conflicts of intention and motivation in the parasite hosts due to their interaction. Accordingly, the hosts coordinate the relative reaction rates of rejection, retrieval, or ignoring to adapt to conflicting intentions and motivations. Both cuckoo parasitism and host defense function as agents for natural selection to adjust the coordination between egg retrieval and rejection adaptations. Unfortunately, studies on this phenomenon are scarce, considerably limiting sufficient comparison and discussion; therefore, we suggest a greater focus on this phenomenon in future studies.

### Supplementary information

Description of Additional Supplementary Files

Supplementary Data 1

Reporting Summary

## Data Availability

All data used in this manuscript is uploaded as Supplementary Data 1.
