# Supplementary material for: Dynamics of evolutionary succession and coordination between opposite adaptations in cuckoo hosts under antagonistic coevolution
Source: Commun Biol. 2024 Apr 3;7:406. doi: 10.1038/s42003-024-06105-9 (PMC10991519; doi:10.1038/s42003-024-06105-9)
Supplement: Supplementary file 1 — Description of Additional Supplementary Files [file 42003_2024_6105_MOESM1_ESM.pdf]

Description of Additional Supplementary Files

File name: Supplementary Data 1
Description: The source data of this study.
